# *Account of Some Discoveries Made by Mr.* Galvani, *of Bologna; with Experiments and Observations on Them. In Two Letters from Mr.* Alexander Volta, *F.R.S. Professor of Natural Philosophy in the University of Pavia, to Mr.* Tiberius Cavallo, *F.R.S.*
*In the Philosophical Transactions these two letters are given in French; for the present translation of them we are indebted to the kindness of a friend.—Editor.


**Published:** 1795

**Authors:** 


XI. *Account of ſome Diſcoveries made by Mr.* Galvani, *of Bologna; with Experiments and Obſervations on them. In two Letters* from Mr.* Alexander Volta, *F.R.S. Profeſſor of Natural Philoſophy in the Univerſity of Pavia, to Mr.* Tiberius Cavallo, *F. R. S.—From the Philoſophical Tranſactions of the Royal Society of London, for the Year* 1793. Part I. 4to. London, 1793.

THE ſubject of the diſcoveries and reſearches, concerning which I am about to write to you, Sir, is *animal electricity*; a ſubject which cannot but be extremely intereſting to you. I know not if you have yet ſeen the work of a Profeſſor of Bologna, Mr. Galvani, which appeared about a year ſince, with this title; ALOYSII GALVANI *de Viribus Electricitatis in Motu Muſculari Commentarius.* Bononiæ, 1791, in 58 pages, 4to, with four large plates; or at leaſt if you have had any

* In the Philoſophical Tranſactions theſe two letters are given in French; for the preſent tranſlation of them we are indebted to the kindneſs of a friend.—EDITOR.

account account of it*. It contains one of the moſt beautiful and ſurpriſing diſcoveries, and the germe of ſeveral others. Extracts from this work have appeared in different Italian Journals, and, among others, in that entitled *Gionale Fiſico-medico*, publiſhed by Dr. Brugnatelli, of Pavia, to whom I myſelf have ſent two long papers, which will be followed by ſeveral others, as I have conſiderably extended my experiments and inquiries on this ſubject. The letters I now addreſs to you are intended as a ſketch both of the admirable diſcovery of Mr. Galvani, and of the progreſs which I have been fortunate enough to make in this new path; and I requeſt you, Sir, to preſent them to Sir Joſeph Banks, Bart. the worthy Preſident of the Royal Society, to be communicated, if he thinks proper, to that learned body, as a feeble teſtimony of my gratitude for the honour they have done me in electing me one of their number, and of my zeal and eagerneſs to comply with their invitation to communicate to them, from time to time, the fruit of my reſearches.

(1.) Mr. Galvani having diſſected and prepared a frog, in ſuch a manner that the legs remained attached to a part of the back bone,

* See Vol. III. p. 180.—Editor.

feparated from the reft of the body, folely by the crural nerves, which were laid bare, obferved that very lively motions were excited in thefe legs, with fpafmodic contractions in all the mufcles, every time that (this part of the animal being placed at a confiderable diftance from the conductor of an electrical machine, and under certain circumftances, which I fhall explain hereafter) a fpark was drawn from this conductor, not on the body of the animal, but on any other body, or in any other direction. The requifite circumftances, therefore, were, that the animal thus diffected fhould be in contact with, or very near fome metal or other good conductor, of fufficient extent, or, what was ftill better, between two fimilar conductors, one of which fhould be turned towards the extremities of the legs of the animal, or fome one of its mufcles; the other towards the fpine, or its nerves: it was likewife very advantageous that one of thefe conductors, which the author diftinguifhes by the names of *conductor of the nerves*, and *conductor of the mufcles*, and preferably the latter, fhould have a free communication with the floor. It was in this fituation efpecially, that the legs of the frog, prepared as above defcribed, received violent fhocks,

ſhocks, ſprang up and contracted with vivacity at each ſpark drawn from the conductor of the machine, although it was at a conſiderable diſtance, and although the diſcharge was made neither on the conductor of the nerves, nor on that of the muſcles, but on any other body, equally remote from them, and having any other communication through which the diſcharge might be tranſmitted, for inſtance, on a perſon placed in the oppoſite corner of the room.

(2.) This phenomenon ſurprized Mr. Galvani, perhaps more than it ought to have done; for the power, not only of electric ſparks when they immediately ſtrike the muſcles or nerves of an animal, but of a current of this fluid traverſing them, in any manner whatever, with ſufficient rapidity, its great power, I ſay, of exciting commotions, was a thing ſufficiently known; beſides, it was obvious how, in this experiment, and in all thoſe of the ſame kind, related in the firſt and ſecond parts of his work, and which are repreſented in the two firſt plates of figures, his frog became liable to be affected by ſuch a current. We have only to conſider that well-known property of electrical atmoſpheres, or what is called *compreſſive electricity*, by which the fluid of conducting bodies, placed within the ſphere of action of an electrified body, is compreſſed and diſplaced, in proportion to the force and extent of this ſphere, and kept in this ſtate of diſplacement ſo long as the electricity ſubſiſts in the predominant body; and when this is removed, returns to its place gradually, if the electricity of that body is ſlowly diſſipated, or in an inſtant if it be deſtroyed inſtantaneouſly, by diſcharging ſuddenly the body that contained it. It is this returning current, therefore, this reflux of electrical fluid in the conducting bodies contiguous to the frog, or near it, its ſudden paſſage from the conductor of the muſcles to the conductor of the nerves, or *vice verſa*, through its body, eſpecially when ſuch a current is compreſſed in the ſingle and narrow channel of the nerves, which excites the ſpaſms and movements in the experiments in queſtion. Mr. Galvani, who ſeems not to have ſufficiently reflected on this property of electrical atmoſpheres, and who was not aware of the prodigious ſenſibility of his frog, ſingularly prepared in the manner above deſcribed (I muſt here obſerve that I have found this ſenſibility nearly equal in all the other ſmall animals, ſuch as lizards, ſalamanders, and mice) was extremely ſtruck with ſuch an effect, which will probably probably not appear ſo marvellous to other philoſophers. This, however, was the firſt ſtep which led him to the grand and beautiful diſcovery of an animal electricity, properly ſo called, and which belongs not only to frogs and other animals of cold blood; but likewiſe to every animal of warm blood, quadrupeds, birds, &c.; a diſcovery which forms the ſubject of the third part of his book, a ſubject altogether new, and very intereſting. It is thus he has opened to us an immenſe field, into which I propoſe to enter, and purſue my reſearches, after I ſhall have dwelt a little more on thoſe preliminary experiments which relate to the action of artificial or extraneous electricity on the nervous and muſcular fibres.

(3.) It was chance that preſented to Mr. Galvani the phenomenon I have been deſcribing, and which aſtoniſhed him (I repeat it) more than it ought to have done. Still who would have believed that a ſtream of electricity, ſo feeble as not to be rendered ſenſible by the moſt delicate electrometer, ſhould be capable of affecting ſo powerfully the organs of an animal, and of exciting in its limbs, cut off one or more hours before, movements, nowiſe inferior in ſtrength to thoſe produced in the living animal, animal, fuch as vigoroufly darting out its legs, fpringing up, &c. to fay nothing of the moft violent tonic convulfions? And yet fuch is the ftream that affects the little animal, placed, for inftance, on a table, near fome metal, or between two good conductors, not infulated, when a perfon draws from the prime conductor, fufpended feveral feet above, a moderate fpark, and conveys the difcharge through quite another channel.

(4.) I fay *moderate*; for if it be very ftrong, and the conductor, large and highly charged, be not at a very confiderable diftance from the bodies on the table, little fparks will be perceptible in the interftices of thefe bodies, efpecially the metallic ones, and even in the place where the frog forms a ring of communication between them, which fparks are evidently produced by the returning ftream of electricity, of which I have already fpoken, (fect. 2.) Or if matters do not come to this point, inftead of fparks we may perceive movements, fufficiently obvious, of electrometers placed on the fame table and in the fame places. In this cafe, therefore, where the electrometer affords the fign, and much more in the other, where the above-mentioned fparks are obtained, we may obferve, that that even a frog, entire and untouched, or any other ſmall animal, as a lizard, a mouſe, or a ſparrow, is ſeized with ſtrong convulſions in all its limbs, eſpecially in its legs, which dart forwards with vivacity, if the paſſage of the electric fluid (the returning ſtream) follows the direction of theſe ſame legs from one end to the other. So far there is nothing wonderful; the circumſtance that may excite ſurpriſe is in the caſe where the ſtream of electricity, though no longer ſenſible, not even to the moſt delicate electrometer, continues to excite the ſame convulſions, the ſame movements, if not in the entire frog, at leaſt in its limbs, when diſſected and prepared in the manner practiſed by Mr. Galvani.

(5.) I have endeavoured, with much attention, to determine what might be the leaſt electrical power requiſite to produce theſe effects, as well in the entire and living frog, as in one diſſected and prepared in the manner above deſcribed, which is what Mr. Galvani has omitted to do. I have preferred the frog to every other animal, becauſe it is endowed with a very durable vitality, and it is very eaſy to prepare it. I have, however, made experiments on other ſmall animals with the ſame view, and with a ſucceſs nearly ſimilar. In order to eſtimate well the the ftrength of the ftream of electricity, I have thought it right to fubmit the animal intended for experiments of this kind, not to the returning ftreams occafioned by electrical atmofpheres (Sect. 2.), but to direct electrical difcharges, fometimes from a fimple conductor, fometimes from a Leyden phial, and in fuch a manner that the whole ftream muft have paffed through the body of the animal. For this purpofe I was careful to keep it infulated in one way or other, and moft frequently by fixing it, with pins, to two flat pieces of foft wood, fupported by glafs columns.

(6.) I have found then, that for the living and entire frog the electricity of a fimple conductor, of a middling fize, is fufficient, when it comes only to be able to give a very weak fpark, and to raife Henley's electrometer from five to fix degrees; that if I make ufe of a Leyden phial, likewife of a middling fize, a much weaker charge of this produces the effect, fuch a one, for example, as yielding not the leaft fpark, and being nowife fenfible to the quadrant-electrometer, is fcarcely fufficiently fo to Cavallo's electrometer to feparate its little pendula about 1-tenth of an inch.

(7.) This, as I have juft now fhown, for a frog entire and untouched; for when it is diſſected and prepared in different ways, and particularly after Galvani's manner, in which the legs are connected with the dorſal ſpine merely by the crural nerves, a much weaker degree of electricity, whether from the conductor or from the Leyden phial (the fluid being obliged to paſs through the narrow paſſage of the nerves), fails not to excite convulſions, &c. Yes, an electricity forty or fifty times weaker, as a charge of the phial that is abſolutely imperceptible to the laſt-mentioned electrometer (Cavallo's), and even to that extremely delicate one of Bennet; a charge, that I was able to render ſenſible only by means of my condenſer, and which I think may be eſtimated at five or ſix hundredths of a degree of Cavallo's electrometer.

(8.) Thus then, in the legs of a frog attached to the ſpine of the back ſolely by its nerves (theſe being laid bare), we have a new ſpecies of electrometer; ſince electrical charges, which from their yielding no ſign to the electrometers already in uſe, would ſeem null, afford ſuch obvious ones to this *animal electrometer*, if I may be allowed the expreſſion.

(9.) When

(9.) When we have feen how, in a frog thus prepared, ftrong convulfions are excited by an extremely weak electricity, by an imperceptible ftream of fluid, we ought furely to be no longer furprifed, that the animal fhould be affected in the fame manner when any body whatever difcharges fuddenly the prime conductor of an electrical machine, and occafions another ftream of electric fluid, great or fmall, of the fluid before difplaced in the conducting bodies near the frog, and which reeftablifhes itfelf, in the manner already explained (Sect. 2.), to pafs rapidly through its nerves. Let us fuppofe this returning ftream to be fcarcely equal to that which a conductor, fufficiently bulky, throws off directly, with an electricity that yields no fpark, and that is almoft infenfible even to Cavallo's electrometer, or a fmall Leyden phial, charged fcarcely a tenth of a degree of this fame electrometer; let us fuppofe, I fay, that the ftream of electricity is not ftronger than this, ftill it will be fufficient, as my experiments, above related (Sect. 6. and 7.), fhow, to excite the movements in queftion.

(10.) But if, after the experiments juft now referred referred to, we ought no longer to be furprifed at thofe of Mr. Galvani, defcribed in the firft and fecond parts of his work, how can we avoid being fo at thofe entirely new and wonderful ones related in the third? Experiments in which he obtained the fame convulfions and violent movements of the limbs, without having recourfe to any artificial electricity, or extraneous excitement, by the fimple application of a conductor, one end of which was made to touch the mufcles, and the other the nerves or fpine of the frog prepared in the manner already defcribed. This conductor, he found, might be either entirely metallic, or compofed partly of metal and partly of other bodies of the clafs of conductors, as water, one or more perfons, &c. Even wood, the walls and floor of the room, might enter into the circle provided they were not too dry; it was only by the interpofition of non-conducting fubftances, as glafs, rofin, and filk, that the effect was prevented. Bad conductors, however, did not do fo well, and only during the firft moments after the animal was prepared, and fo long as the vital powers remained in full vigour; after which good conductors only were found to fucceed, ceed, and in a ſhort time it was found impoſſi-ble to produce the effect unleſs with excellent conductors, that is, with conductors entirely metallic. He moreover found a great advantage from applying a ſort of metallic armour, or coating, to that portion of the ſpine which he left attached to the crural nerves, and to the nerves themſelves, and particularly from covering this part with a thin leaf of tin or lead.

(11.) Mr. Galvani did not confine himſelf, in theſe truly aſtoniſhing experiments, to frogs; he extended his trials with ſucceſs, not only to ſeveral other animals of cold blood, but likewiſe to quadrupeds and birds; in all of which he obtained the ſame reſults, by means of the ſame preparations, which conſiſted in laying bare ſome principal nerve at the part where it paſſes into a limb ſuſceptible of motion, and after arming the nerve with ſome metallic ſubſtance, forming a communication, by means of his conductor, between this coating and the muſcles to which the nerve is diſtributed.

(12.) It was thus he fortunately diſcovered, and demonſtrated to us, in the moſt evident manner, the exiſtence of a real *animal electricity* in all, or almoſt all animals. It ſeems in fact to be proved by his experiments, that the electric fluid fluid tends inceſſantly to paſs from one part to another of a living organized body, and even of detached limbs, ſo long as any remains of vitality ſubſiſt in them; that it tends to paſs from the nerves to the muſcles, or *vice verſa*, and that the muſcular movements are owing to a ſimilar transfuſion, more or leſs rapid. In truth, it would ſeem that no objections can be raiſed to this, or to the manner in which Mr. Galvani explains it, by a kind of diſcharge ſimilar to that of the Leyden phial. But a great number of new experiments that I have made on this ſubject, will ſerve to ſhow that many reſtrictions muſt be made with regard both to the thing itſelf, and to the deductions the author has drawn from it; my experiments likewiſe will be found conſiderably to extend the phenomena attributed to this animal electricity, and will diſplay it to us under a great number of new circumſtances and combinations.

(13.) Mr. Galvani, purſuing the idea he has formed to himſelf from his experiments, and adhering in every reſpect to the ſuppoſed analogy of the Leyden phial, and his conductor, imagines there is naturally an exceſs of electric fluid in the nerve, or in the interior part of the muſcle, and a correſponding defect of this fluid fluid in the outer part, and *vice versa*; and he suppofes confequently that one end of this conductor muft communicate with the nerve, which he confiders as the conducting wire or hook of the phial; and the other end with the external furface of the mufcle. All the figures of his third and fourth plates, and all his explanations relate to this. But if he had a little varied his experiments, as I have done, he would have feen that this double contact of nerve and of mufcle, this circuit which he imagines, is not always neceffary. He would have found, as I have, that the fame convulfions, the fame movements may be excited in the legs and other limbs of frogs, and of every other animal, by placing metallic fubftances in contact with two parts of a nerve only, or with two mufcles, or even with different parts of a fingle and fimple mufcle.

(14.) It is true we are very far from fucceeding fo well in this way as in the other, and that in this cafe it is neceffary to have recourfe to an artifice, of which we fhall have occafion to fpeak more fully hereafter, and which confifts in employing two different metals; an artifice, which is not abfolutely neceffary when the experiment is conducted according to Galvani's method method above defcribed (Sect. 10 and 11), at leaft fo long as vitality remains in full vigour in the animal, or in its detached limbs; but, at any rate, fince by arming the nerves only, or the mufcles only, with different metals, we are able to excite contractions in the latter, and movements in the limbs, we muft conclude that if there are cafes (and this may perhaps ftill be very doubtful) where the pretended difcharge between nerve and mufcle (Sect. 12 and 13.) is the caufe of the mufcular movements, there are likewife many and more frequent circumftances, where the fame movements are obtained by quite another play, quite another circulation of the electric fluid.

(15.) Yes, it is quite another play of the electric fluid, of which we may be faid rather to difturb than to reftore the equilibrium, info-much as it paffes from one part to another of a nerve, a mufcle, &c. as well internally by their conducting fibres, as externally by means of the metallic conductors that are applied, not in confequence of any refpective excefs or defect, but by a peculiar action of thefe fame metals, when they are of different kinds. It is thus I have difcovered a new law, which is not fo much a law of animal electricity as a law of common electricity; to which we muſt attribute the moſt part of the phenomena, which, from the experiments of Galvani, and from ſeveral others which I made myſelf, ſeemed to belong to a true ſpontaneous animal electricity, but which in truth do not: they are really the effects of a very feeble artificial electricity, which is excited in a way never before ſuſpected, by the ſimple application of two coatings of different metals, as I have already hinted, and which I ſhall explain better elſewhere.

(16.) I think it right here to ſay, that at the diſcovery of this new law, of this, till now, unknown artificial electricity, I was miſtruſtful of every thing that ſeemed to me to demonſtrate a natural electricity, in the ſtrict ſenſe of the term, and that I was on the point of giving up this idea. But upon carefully reconſidering all the phenomena, and repeating the experiments under this new point of view, I found that ſome of them ſupport ſuch an idea, (thoſe, for inſtance, in which there is no need of different coatings, or even of any coating, a ſimple metallic wire, or any other conducting body, performing the office of conductor between the nerve, and one of the muſcles connected with it, being capable of exciting convulſions in the latter), latter), (Sect. 10, &c.) and that thus a natural animal and properly organic electricity fubfifts, and cannot be entirely overturned. The phenomena which eftablifh it, although much more limited, are however fufficient to demonftrate its exiftence, as I have juft now mentioned, and as will more clearly be fhown hereafter.

(17.) What will perhaps be found more difagreeable is, that we muft likewife confine within narrower limits its influence in the animal œconomy, and give up the fineft ideas we had formed of it, and which feemed to be about leading us clearly to explain mufcular motion. My experiments, varied in every manner poffible, fhow that the motion of the electric fluid excited in organs, does not act immediately on the mufcles; that it does nothing more than excite the nerves, and that the latter, put into action, excite in their turn the mufcles. What this action of the nerves is; how it propagates itfelf from one part of a nerve to another; how it paffes to the mufcles, and how the motion of the latter refults from it; thefe are problems, in the explanation of which we are not farther advanced than before the difcovery in queftion.

(18.) I come now to the experiments that prove all the affertions I have advanced in thefe last paragraphs. From a great number I shall select only a few, which seem to me the best calculated to establish certain principles, for the most part new and different from those adopted by Mr. Galvani. But I must first say a few words more concerning the experiments of this writer. I know not whether he has made others, but those he has described in his work are included in too narrow a circle; in all of them the object is to lay bare and insulate the nerves, and to establish a communication, by means of conducting bodies, between these nerves and the muscles that are dependent on them (as may be seen in all the figures of the four plates annexed to his work), in order to excite convulsions and movements of the muscles, by the action of the electric fluid. He supposes therefore, in every case, and he explains himself pretty clearly on this point, that the transfusion of the electric fluid that is produced, whether by artificial electricity, or by natural animal electricity, must take place from the nerves to the muscles, or *vice versa*; that these two limits at least must be included in order for the muscular movements to take place; and in truth all the experiments he has described seem to prove this. But then they are confined, as I have just now

 said,

ſaid, within a circle that is too limited, and beyond which he has never, or ſcarcely ever, extended his inquiries. By varying the experiments of this kind in different ways, I have ſhown, that neither the one nor the other of thoſe conditions, viz. the laying bare and inſulating the nerves, and the touching ſimultaneouſly theſe and the muſcles, in order to procure the ſuppoſed diſcharge, are abſolutely neceſſary (Sect. 13.). It is ſufficient, when, for inſtance, we have laid bare the iſchiatic nerve of a dog, lamb, &c. if we paſs a ſtream of electricity from one part of this nerve to the other, even though it be near, and leave all the reſt untouched and free; it is ſufficient, I ſay, to do this in order to excite in the limb very ſtrong convulſions and movements; and this whether we employ an extraneous artificial electricity, or excite the electric fluid that is inherent in the nerve itſelf. Here is the manner in which I make theſe experiments.

(19.) Experiment A. I compreſs, with a pair of forceps, the iſchiatic nerve a little above its inſertion into the thigh, and I apply, a few lines higher up, a piece of money, or a plate of metal, on this ſame nerve, carefully ſeparated from the parts that adhere to it, and ſupported by a thread, a plate of glafs, a ftick of fealing wax, a piece of dry wood, or any other fubftance that is a bad conductor. Then placing the belly of a Leyden phial, very weakly charged, on the forceps, I bring the hook into contact with the other piece of metal; and the moment the difcharge takes place, although it be too feeble to produce the leaft fpark, convulfions take place in all the mufcles of the thigh and leg, the whole limb being agitated and fpringing up with more or lefs violence. And yet the whole of this leg, and even a part of the nerve which paffes to it, are, as we fee, out of the track which the electric fluid takes in its paffage, fo that only a fmall portion of the nerve can have been irritated; and yet this is fufficient to occafion the convulfion of the mufcles.

(20.) Experiment B. The fame effects, that is to fay, fimilar convulfions and motions of the leg, take place, without our having recourfe to an extraneous electricity, by the difcharge which takes place, in a certain manner naturally, when we apply, as above, the fame forceps, or a plate of filver, to one part of the nerve, and a plate of fome other metal, and above all, of tin or lead, to another part, and then bring about a fimple communication between tween them, either by an immediate contact, or by the interposition of a third piece of metal made to perform the office of a conductor.

(21.) Thus we see that the same effects, that is, convulsions and violent muscular contractions, take place without any discharge of electric fluid between the nerves and muscles, in the manner Mr. Galvani supposes; and without requiring one end of a conductor to communicate with the one, and the other end with the other. Neither is the other condition, that of laying bare the nerve, and freeing it of its adhesions, at all more necessary, as will appear from the following experiments.

Experiment C. I apply coatings, or plates, of different metals, (and it is this difference of coatings that is essential) (Sect. 14. and 15.) to an entire and living frog, that is covered with its skin, and, in short, is untouched. I apply, for example, a thin piece of tin-foil on its back, or its loins, and I place a piece of silver money under its thighs, or its belly, slightly compressing it; this done, I slide forward the piece of money till it comes into contact with the tinfoil, or I form a communication between the two metals by means of a piece of iron wire, or any other metal; and at that instant convulsive motions take place in all the mufcles of the belly, thighs, and back, with violent tremors of the legs, contraction and curvature of the fpine, &c. which convulfions and fpafms, although nearly univerfal, are however moft confiderable in the limbs and mufcles contiguous to the coatings, and ftill more fo in thofe which are dependent on the principal nerves neareft to the two metals.

(22.) Thefe experiments fucceed in fome other animals; in fifhes, and particularly in eels, in none of which is it neceffary to remove the fkin, though it does not fail, in a fmall degree, to leffen the effect. This is why, by removing it, at leaft in part, particularly in the frog, we obtain the effects with more certainty, and to a greater degree. We likewife gain fomething, in this refpect, by cutting off the head of the frog, and thrufting a large pin into the fpinal marrow; we then excite, by means of different coatings in the manner above defcribed, ftronger movements, or at leaft fuch as are more obvious, becaufe they are no longer confounded with the movements the animal gives itfelf while living.

(23.) If it be advantageous, as we have feen, to take off the fkin of frogs, although very thin and and pretty moiſt, it is much more ſo, and even neceſſary, to remove it from almoſt all the other animals, as lizards, ſalamanders, ſerpents, tortoiſes, and more eſpecially from quadrupeds and birds, that are furniſhed with a drier and much thicker ſkin, to ſucceed in theſe experiments. The following, therefore, is the mode I adopt.

Experiment D. I faſten to a table, by means of ſome large pins, a lizard, a mouſe, a fowl, &c. and after making an inciſion through the ſkin, and other integuments, to the bare fleſh, on the back of the animal, I turn back the integuments on each ſide; I do the ſame on the thigh or the leg; after which I apply the two metallic coatings on the expoſed parts, viz. on one the tin foil, and on the other a ſpoon or a piece of money; I then form a communication between the two coatings, and every time I do this I excite ſtrong contractions in the adjacent muſcles, and particularly in thoſe of the thigh and leg, which moves and agitates itſelf with great violence. Theſe convulſions are much more conſiderable when the tin foil is applied near the iſchiatic nerve, and the piece of ſilver on the gluteus muſcle, or on that named gaſtrocnemius; and the effects are ſtill greater if the nerve itſelf is laid bare, and coated with the the tin foil; if, leaving it attached only to the mufcles to which it is diftributed, we deprive it of every other adherent part; or if, in fhort, we feparate the entire limb from the reft of the body, with its nerve hanging out, and fubmit it in this ftate to our experiments.

I am, &c.

A. VOLTA.

*September* 13, 1792.

---

### SECOND LETTER.

(24.) It will be fufficiently underftood that what I have faid with refpect to the ifchiatic nerve, and the leg, is applicable to the brachial nerve and to the arm, as well as to every other nerve relatively to the mufcles under the influence of that nerve.

(25.) Thefe laft preparations are analogous to thofe of Mr. Galvani; and they clearly prove that it is advantageous to lay bare the nerves, and ftill more fo, to detach them all round from the adherent parts; but they are far from fhowing ing that this is a neceſſary condition, ſince we never fail to obtain the ſame convulſions and movements of the limbs when we ſimply lay bare the muſcles, and leave the nerves covered and concealed under them in their natural ſtate, as all my other experiments above related (Sect. 21, 22, 23.) ſerve to ſhow.

(26.) After theſe trials on reptiles, birds, and ſmall quadrupeds, I proceeded to other and larger animals, as rabbits, dogs, lambs, and bullocks; and I not only ſucceeded in obtaining ſimilar effects in all the ways above deſcribed, but even ſtronger and more durable ones, by reaſon that the vital heat maintained itſelf in thoſe large animals, and in their limbs, a longer time. For I ought not to omit to ſay, that if in the moſt part of animals of cold blood, and particularly in frogs, the vital principle ſubſiſts in detached limbs ſeveral hours, that principle which renders them ſo ſenſible to the weakeſt electrical irritation, it hardly continues beyond a few minutes in animals of warm blood, and commonly diſappears before the whole of this animal heat is diſſipated.

(27.) Having had ſuch ſucceſs with my experiments on large and ſmall animals of every kind, in ſome inſtances alive and entire; in others others deprived of their ſkin, or their head, or diſſected in different ways ; and having obtained ſimilar effects in their large detached limbs, and almoſt always without the preparation required by Mr. Galvani, that is to ſay, without laying bare the nerves, I was deſirous of going ſtill farther, and of making ſimilar trials on ſmaller limbs, on a ſingle muſcle, and even on ſmall portions of muſcles ; and the freſh ſucceſs I had in theſe trials led me to other diſcoveries, which I will ſoon mention, after having deſcribed ſome of theſe experiments.

(28.) Experiment E. I cut off, in ſome inſtances, the leg and thigh of a frog, in others, the leg only, and in ſome half or a quarter of a leg ; and on applying, as uſual, to one part of the amputated portion the tin foil, and to the other the plate of ſilver, and forming a communication between theſe two coatings, I conſtantly excited convulſions and movements; I have even ſeparated a ſingle muſcle, for inſtance the gluteus, or the gaſtrocnemius, and ſometimes only a portion of muſcle not larger than a barley-corn, and yet the ſame effects, that is to ſay, very ſtrong contractions of theſe muſcles, or parts of muſcles, have been produced by means of two different coatings, &c.

Expe-

EXPERIMENT F. I have repeated the fame experiments on a leg, on a half or a third part of the leg, on a fingle mufcle, or part of a mufcle, of a fowl and other birds; on a flice of the gluteus of a rabbit, a lamb, &c. and I have had the fame effects as long as the flefh preferved a fenfible heat. (Sect. 26.)

(29.) Thus then we are able to excite very ftrong contractions in the mufcles of animals of warm as well as of cold blood, and in every detached portion of mufcular flefh; and this by means of the fimple artifice of different metallic armours or coatings, applied to the mufcle itfelf, without any preparation of the nerves, and even without laying them bare. We have befides feen that we can excite thefe effects quite as well, and by the fame means of metallic coatings applied to two neighbouring parts of the fame nerve, (Sect. 19, and 20. Experiments A. and B.) whence I have reafon to conclude, that there is no neceffity for a difcharge of electric fluid to take place between nerve and mufcle, or for any tranfmiffion of it from the interior to the exterior part of the latter by means of the nerve and metallic conductor, as Mr. Galvani fuppofes, or *vice verfa:* and that there is no comparifon to be made between tween the muſcle and the Leyden phial and its diſcharge, in the experiments in queſtion. In fact, what reſemblance or analogy is there to the Leyden phial, where the two plates of metal, a communication between which is formed by the conductor, are applied very near to each other on the external ſurface of the ſame nerve, (Experiments A. and B.) or on the external ſurface of two muſcles, or even of the ſame muſcle (Experiments C. D. E. F.); it muſt be conſeſſed it would be in vain to attempt to ſupport any analogy between any of theſe experiments and the Leyden phial.

(30.) Experiment G. Having placed two coatings, one of ſilver leaf, the other of tin foil, on exactly correſponding parts of the two thighs of a frog, I excited contractions of the muſcles, and the uſual motions of the legs, at the inſtant I formed a communication between the two coatings by means of the conductor.

(31.) Is it thus, I aſk, that the diſcharge of two Leyden phials takes place, by forming a communication between their homologous ſurfaces? Let us lay aſide, therefore, theſe ideas of phial and diſcharge, and every forced explanation, and let us ſay ſimply, that in theſe and other analogous experiments, a tranſmiſſion of the electric fluid takes place from one to another of two parts properly coated; a transmission determined, not by a *relative excess* of this fluid, which cannot naturally be supposed between parts that are similar, but by the diversity of these same coatings, which must be of different metals, as I have taken care already to point out, (Sect. 20, and 21. Experiments B. and C.) and uniformly to inculcate in the subsequent parts of my paper.

In fact,

(32.) EXPERIMENT H. If two muscles, or two parts of the same muscle, are similarly coated, that is, with two plates of the same metal, both of them equal in temper and hardness, in softness or rigidity, in the roughness or smoothness of their surface, and both are applied in the same manner, it will be to no purpose to bring about a communication between them by means of a conductor, as no convulsion, no motion will take place.

(33.) I confess it is not easy to conceive how and why the simple application of two dissimilar coatings, I mean of two different metals, to similar parts of the animal, and even to two parts very near to each other of any one muscle, shall disturb the equilibrium of the electric fluid, fluid, and drawing it from its ftate of repofe and inactivity, fhall induce it to pafs inceffantly from one part to another; which transflux takes place as foon as a communication, by means of the conductor, is formed between thefe two diffimilar coatings, and continues all the time this communication fubfifts. But conceivable or not, and whatever may be the caufe, it is a fact that the experiments I have already related fufficiently prove, and which will be confirmed by many others, to the defcription of which I fhall endeavour to add fome explanation. It is a fact, to be added to what we already know in electricity; a fact which muft furely appear extraordinary, and difficult to be reconciled with the laws commonly eftablifhed. It is truly a new and very fingular law, which I have difcovered; a law that belongs not properly to animal electricity, but to common electricity, fince this transflux of the electric fluid, a transflux, not momentary, as a difcharge would be, but which continues as long as the communication between the two coatings fubfifts, and takes place whether thefe coatings are applied to living or dead animal fubftances, or to other conductors not metallic, but fufficiently good, as water, or moift bodies. But before I proceed to the experiments periments which decifively prove all that I have advanced, I think it right to offer a few more remarks on thofe I have already defcribed (Sect. 20—32.).

(34.) It would feem from thefe that by means of the fimple artifice of coatings of different metals fuitably applied, we are able to excite very ftrong convulfions in every mufcle of every animal, fo long as it continues to poffefs any degree of vitality. Such a conclufion, however, would be too general, my experiments having taught me that it is to be admitted only with certain reftrictions, as well with refpect to the claffes and genera of animals, as with refpect to the different mufcles of each animal.

(35.) And firft with refpect to the different claffes of animals; although it has uniformly happened that all the quadrupeds, birds, fifhes, reptiles, and amphibious animals, which have been fubmitted to my experiments, exhibited the phenomena above defcribed, it is no lefs certain that worms in general, and feveral fpecies of infects, remained unaffected. I have in vain tried with worms, leeches, fnails, oyfters, and different caterpillars; I have not even been able to excite the leaft motion in them by fmall and moderate fparks, and difcharges of artificial

 electricity.

electricity. Here is the manner in which I proceeded.

Experiment I. I applied the tin foil, and silver leaf, to different parts, as well external as internal, of these snails, leeches, earth worms, &c. and in the best way I was able; I then formed a communication between these metallic coatings, sometimes by bringing them into contact with each other, and at others by means of another metal that performed the office of a conductor; but by neither of these means could I ever obtain the least motion in any part of the body.

Experiment L. I conveyed through their bodies, both when insulated and not insulated, discharges of a Leyden phial of sufficient strength to excite a moderate spark, and to give me a slight shock, but they were not sensibly affected by it; no motions or convulsions were produced.

(36.) Does it follow from hence that the more imperfect animals, the whole class of worms, and several species of insects, are destitute of that sensibility and irritability, that electrical mobility, if I may be allowed the expression, with which other more perfect animals are endowed? I am unwilling to draw this general conclusion from my experiments, because I have as yet extended them only to a ſmall number of worms and inſects; and with regard to the latter, I think it right to obſerve that I have ſucceeded, without much difficulty, with craw fiſh, beetles, graſshoppers, butterflies, and flies. It may not be uſeleſs that I explain one of the ways in which I ſucceed with theſe animals, as they are with difficulty ſubmitted to experiments, on account of their minuteneſs, or of the ſcales with which they are covered.

Experiment M. After cutting off the head of a fly, a butterfly, beetle, &c. I ſlit open, with a penknife or ſmall ſciſſars, the whole length of the corſlet, and introduce deep into the ſlit, near the neck, a bit of tin foil, (what is improperly called ſilver paper is very fit for this purpoſe) and a little below I introduce, and likewiſe deep into the ſlit, a bit of ſilver plate, or ſmall ſilver coin; and when I bring the latter into contact with the piece of tin foil, the legs begin to bend and tremble, and the other parts, and even the trunk of the animal, are thrown into agitation. It is very amuſing to excite in this manner the chirping of a graſshopper, &c.

(37.) After what I have juſt now ſaid, I ſhould be wrong to rank inſects among the animals that are deſtitute (like the claſs of worms

 above above mentioned) of the electrical property in queftion. At the utmoft, if caterpillars appear to be fo, it may be faid that in this ftate of larva, before they have attained, by their metamorphofis, a perfect ftate, and acquired new organs, &c. they may be compared in many refpects to worms, and, like thefe, are not endowed with electric fenfibility.

(38.) In fhort, if I may be allowed to ftate here what I think, thofe animals only that have very diftinct limbs, with joints, and mufcles fitted for the motion of thofe joints, or, in other words, mufcles that are called flexors, or levators, and nerves proper to regulate them, fuch animals only, I fay, are fenfible to, and become feized with real fpafmodic contractions in confequence of either fmall difcharges of artificial electricity, or a weak current of fluid occafioned fimply by different metallic coatings; which contractions and fpafms bring on the motion, and even a violent agitation of the faid limbs. On the contrary, worms, and fuch infects as have not fufficiently diftinct limbs, or joints properly fo called, or which are deftitute of flexor mufcles, or enjoy only a vermicular motion, are nowife affected by fuch an electricity. The motions of thefe animals depend on a different a different animal œconomy; on a different mechanifm, which in feveral fpecies has been very well difcovered and explained. Such are my ideas, ftill indeed fomewhat vague, and founded only on a few experiments; it is the fequel of thefe that muft either confirm or rectify them.

(39.) With refpect to different mufcles in the fame animal, I am able to advance fomething more certain. I fay then, that all mufcles are very far from being fufceptible of contraction from the weak electricity in queftion. There is a great diftinction to be made with regard to their functions in the animal œconomy; all of them are not fubject to the empire of the will, and fitted for fpontaneous movements: and, ftrictly fpeaking, it is only thofe which are fo that are capable of fpafmodic contractions by the means above defcribed; yes, the mufcles fubject to the will are the only ones I have found fufceptible of irritation, and motion, by the action of that weak current of electric fluid occafioned by the fimple contact of two different metals. The other mufcles, over which the will has no direct power, as thofe of the ftomach, inteftines, &c. are not at all fo, not even the heart, though in other refpects fo irritable. We muft except, however, the mufcles of the diaphragm, (and I conjectured it before I made the trial) these being of the number of those whose motion depends on the will.

Experiment N. It is very surprising that a slice of good muscular flesh, cut, for instance, from the thigh of a lamb killed half an hour or an hour before; that this piece, I say, of muscle, almost quite cold, and which is no longer sensible to the action of any mechanical or chemical stimulus, should be so powerfully affected by the electric fluid conveyed from one part of it to another, as to be seized with very strong spasmodic contractions; and that, on the contrary, the heart recently taken out of the same animal, and still warm and very irritable, should, when treated in the same manner, with the best adapted metallic coatings, suffer no alteration upon our making a communication between the two metals by means of the conductor; and that its pulsations, when weakened or slackened, or altogether suspended, should not be increased, or even revived, notwithstanding all this takes place from the application of the slightest mechanical or chemical stimulus.

(40.) The electric fluid, therefore, which seems to be the stimulus appropriated to the muscles of the will, is nowise so to the heart, or or to the other mufcles formed for involuntary vital and animal functions. But what will be faid if I make it appear that it is not the immediate or efficient caufe of motion in the voluntary mufcles; that even in thefe it is a mediate caufe only, the nerves alone being directly affected by it? And yet this is what I have learned from feveral experiments; experiments that have obliged me to give up the fineft and moft extenfive ideas I had formed on the fubject. I was fond of thinking, with Mr. Galvani, that as often as a current of the electric fluid, put in motion in the organs, was impelled with a certain degree of ftrength to the mufcles, this fluid did itfelf perform the office of a ftimulant, and excited the irritability which is peculiar to them; that every mufcular movement was executed in confequence of a fimilar irruption of electrical fluid into the mufcles, either by means of artificial electricity, or by 'putting in motion the natural artificial electricity; that, in fhort, even the motions which are performed naturally in the living animal machine, at leaft the voluntary motions, acknowledged the fame caufe, that is to fay, the immediate action of the electric fluid on the mufcles. But I repeat it, I have found myfelf obliged, with regret, to give up all those fine ideas by which it seemed possible to explain things to admiration. Yes, we must considerably limit the action of electricity in animals, and consider it under another point of view, that is to say, as being capable of exciting, of itself, the nerves, as I have already hinted, and as I shall now proceed to prove.

(41.) In the first place, then, that it can act, and that it really does act, on the nerves, and that the latter, excited by it, excite in their turn the muscles connected with them, without even the electrical stream's arriving at those muscles, is a fact which no longer stands in need of proofs after those furnished by the experiments A. and B. (Sect. 19. and 20.) and even by an experiment of Mr. Galvani, which, according to his account, was the first he made, and the origin of all his other experiments. It is sufficiently obvious that the electric current, in the experiment in question, as well as in those made by me, and which I have just now referred to, pervades only a part of the crural nerve, but not one of the muscles of the leg; and yet as the latter depend on the nerve, they are affected with convulsions.

(42.) But I go farther, and maintain, that even in the cafes where the electrical current (it will be clearly underftood that I am fpeaking only of weak artificial difcharges, or of the current which takes place by the fimple application of coatings of different metals) ftrikes and penetrates mufcles fufceptible of movement, it is not by irritating the latter immediately that it occafions them to contract, but by ftimulating their nerves. This is what is fhown by my experiments C. and D. (Sect. 21. and 23.) where, upon the tin foil and piece of filver being applied immediately to the mufcular parts of the animal, whether the animal or only a detached portion of it is the fubject of the experiment, it is not fo much the mufcles covered by the two metallic coatings that fuffer the moft violent contractions, as thofe which depend on fome principal nerve, to which one or other of the coatings is contiguous. It is in this manner that in the frog, when the tin foil is applied on the loins, where the crural nerves lay at but little depth, the mufcles of the legs are feized more than any others with ftrong convulfions, more fo even than thofe contiguous to the other coating, that is to fay, to the piece of filver. I have already pointed out the fame thing in quadrupeds, dogs, lambs, &c. with regard gard to the iſchiatic nerve, (Experiment D.) and I have only to add, that the leg never fails to be convulſed when this nerve does not lay too deep under the fleſh and other integuments, and one of the coatings is properly applied to this part; even although the other coating ſhould be made to correſpond neither with the gluteus nor any muſcle of the leg, but with any other muſcle whatever, provided it be not at too great a diſtance. Here is another proof why this happens:

Experiment O. If we apply in a frog, or any other ſmall animal, the tin foil the whole length of the ſpine of the back, from which proceed all the nerves of the trunk and limbs, and the other coating to any other part whatever, all the limbs become affected; the muſcles, not only of the legs, but of the belly and back, experience ſpaſmodic contractions, and the trunk itſelf becomes curved; in a word, the convulſions are general. The experiment is ſtill more ſtriking in a lizard than in a frog, and I ſhall therefore deſcribe it.

Experiment P. After cutting off the head of a lizard, and laying bare the muſcles of the back by removing the ſkin, I apply a piece of tin foil to the mutilated end, in ſuch a manner that that the tin foil is ſpread beyond the edges of the wound, ſo as to riſe a little over the ſhoulders, and I place a piece of money on the middle of the ſpine; this done, I ſlide forward the piece of money till I bring it into contact with the tin foil. At that inſtant the legs move, the tail twiſts itſelf, and the whole body of the animal becomes agitated, and darts from right to left, and from left to right. Is not this becauſe the upper part of the ſpinal marrow, the principal ſource of the nerves, is irritated?

(43.) Nearly the ſame effects may be obtained by a ſimilar operation on a mouſe, a ſmall bird, &c. but in theſe it is neceſſary to remove not only the ſkin and other integuments, but likewiſe ſome of the fleſh, and this becauſe their back being more fleſhy, the principal nerves of the ſpine are more concealed by this fleſh, and by the bones alſo of the vertebral tube. It is in fact eaſy to comprehend that the current of electric fluid, occaſioned by the two coatings, penetrating only to a certain depth the parts of the animal covered by theſe coatings, can hardly reach the ſpinal marrow, or the principal branches of the nerves that enter into the interior parts of the limbs, if the bones, fleſh, and other intervening integuments are of con-

ſiderable

ſiderable thickneſs. The reaſon alſo muſt be obvious, why, in the larger animals, as dogs, lambs, &c. we fail to excite contractions in all the limbs by the application of the two coatings to the back, although ſtripped of its fleſh. The large trunks of the nerves remain ſtill at too great a depth; and it is only the ſmaller branches or ramifications that lay but a little below the coatings, and theſe branches terminate, for the moſt part, only in the neighbouring external parts; conſequently we ſee produced only ſuperficial contractions or palpitations in one or other of the muſcles: or if by chance a whole limb is put in motion, it is becauſe the nerve that goes to it, and influences this motion, is but thinly covered, ſo that only a thin layer of fibres intervenes between it and one or other of the metallic coatings, as appears from Experiment D. and the following ones (Sect. 23. &c.) in which the application of one of the coatings near the iſchiatic nerve, in a dog or a lamb, was ſufficient to excite conſiderable movements in the leg; and the nearer the coating was to the nerve, and the thinner the layer of fleſh was that ſurrounded it, ſo much ſtronger in proportion were the contractions of the limb.

 (44.) It

(44.) It becomes therefore neceſſary to know the ſituation of the nerves, their direction, &c.; and it is requiſite to remove not only the common integuments, the fat, &c. but likewiſe part of the fleſh that covers and ſurrounds the nerves, in order that this ſurrounding muſcular ſubſtance may be more or leſs extenuated, previouſly to the application of the metallic coating, to enable us to obtain in the larger animals contractions in any particular limb, to ſay nothing of the ſuperficial contractions and palpitations of one or more muſcles. It is perhaps impoſſible to excite theſe ſame motions and contractions in all the limbs at once; although this is not difficult in the ſmaller animals, as we have already ſeen, (Sect. 42. Experiments O. and P.) merely by depriving them of the ſkin or a part of the other integuments; and even this is not neceſſary in frogs, for in theſe animals we may leave the ſkin, it being ſo extremely thin and moiſt, as not to prevent, by its interpoſition, the electrical current from reaching the principal nerves or the ſpinal marrow.

(45.) But if it be neceſſary to pay attention to the direction of the principal nerves, in order to bring on the contractions in the different limbs, it is not leſs ſo to be careful of the poſition fition of the coatings relatively to the mufcles; for thofe mufcles which are neareft to one or other of the coatings, are in general the moft liable to contract fpafmodic convulfions, and are oftentimes the only ones in which fuch an effect takes place; as, for inftance, when the coatings do not correfpond with any confiderable nerve, or if there be a nerve, when it is furrounded with too much mufcular flefh, or is too deeply feated.

(46.) This, and the Experiments E. F. (Sect. 28.) where a fingle mufcle, and even a part of a mufcle, treated in the ufual way, experienced very ftrong contractions, might lead to a fuppofition that the electric fluid produces thefe effects by irritating the mufcular fibres themfelves, without the intervention of nerves; the action of which would confequently be neither *primary*, nor abfolutely neceffary, as I pretend. But an argument of this fort, founded on thefe facts, can have no weight, unlefs it could be proved that in thefe mufcles, or portions of mufcles, there are no nerves; for if there are nerves, (and certainly there muft be, and are, nervous filaments in every fenfible portion of a mufcle, I had almoft faid in every mufcular fibre) I may ftill maintain that it is thefe nervous filaments, ramifying through the whole ſubſtance of a muſcle, that are immediately affected by the electric fluid which penetrates this ſame ſubſtance; that this fluid exerting its influence on their nerves, an influence that finiſhes there, the latter exert theirs on the muſcles, &c. I may, I ſay, be able to maintain, with ſufficient probability, that the electric fluid has no other influence, in the phenomenon of muſcular contractions, than that of exciting the nerves; in a word, that it is not the immediate cauſe. Such an aſſertion, which the things already explained render more than probable, is proved directly, and in the moſt obvious manner, by ſeveral experiments I have made on the tongue; experiments that have led me to other diſcoveries equally intereſting and curious.

(47.) Having ſucceeded in exciting tonic convulſions, and the moſt violent motions in the muſcles and limbs, not only of ſmall but of large animals, without laying bare any nerves, by the ſimple application of coatings of different metals to the muſcles when freed from their integuments, I ſoon thought of trying whether the ſame effects might not be obtained in the human body. I conceived that the thing might ſucceed very well in amputated limbs; but in the entire tire and living ſubject how was it to be effected? It ſeemed likewiſe to be neceſſary to remove the integuments, make deep inciſions, and even diſſect off portions of the fleſh from the parts on which we might think of applying the metallic coatings (as I have remarked we are often obliged to do in the larger animals). Fortunately it came into my head, that we have, in the tongue, a muſcle that is bare, or at leaſt deſtitute of thoſe thick integuments with which the external parts of the body are covered, a muſcle which is extremely moveable, and moveable at will. Here then, I ſaid to myſelf, are all the conditions requiſite to enable us to excite movements by the uſual artifice of different metallic coatings. With this view I made, on my own tongue, the following experiment.

(48.) Experiment Q. Having covered the point of the tongue, and a part of its upper ſurface, to the extent of ſome lines, with a piece of tin foil, (what is called ſilver paper is the fitteſt for the purpoſe) I applied the convex part of a ſilver ſpoon farther on, on the flat part of the tongue, and by inclining the ſpoon downwards brought the handle of it into contact with the tin foil. I expected to ſee my tongue affected with tremor; and on this account count I made the experiment before a looking-glafs. The effect, however, I had ventured to foretel did not take place; but inftead of it I had a fenfation I nowife expected; this was a pretty ftrong acid tafte on the point of the tongue.

(49.) I was at firft much furprifed at this; but upon reflecting a little on the fact, I eafily conceived, that the nerves which terminate on the point of the tongue, being the nerves deftined for the fenfations of tafte, and not for the motion of this flexible mufcle, It was perfectly natural, that the irritation of the electric fluid, put in motion by the ufual artifice, fhould excite a tafte, and nothing more; and that in order to excite in the tongue the motions of which it is fufceptible, it would be neceffary to apply one of the metallic coatings near its root, where the nerves enter that influence its motion; and this I foon verified by another experiment, as follows:

(50.) Experiment R. Having cut out, from a lamb recently killed, the tongue near its root, I applied a piece of tin foil at the end that was cut, and the filver fpoon to one of the furfaces of the tongue; and then forming a communication between thefe two metallic coatings, I had the pleafure to fee the whole tongue affected with tremor, raifing its point, and turning and bending itfelf in different directions, every time, and as long as fuch a communication took place.

(51.) I have repeated this experiment on the tongue of a calf, which I placed, coated in the fame manner with a piece of tin foil near its root, on a filver plate, that the latter might ferve as another coating; and the fuccefs was the fame. I have likewife repeated it on the tongue of other fmaller animals, as mice, chicken, rabbits, &c. and I have almoft always obtained the fame effect. I fay *almoft always*, for in the tongue of the fmaller animals it fometimes failed; either becaufe the tin foil was not applied exactly to the proper place, where the nerves that influence the motions of the tongue are inferted; or becaufe the tongue being cold, had loft its vitality, which feldom lafts long in the mufcles of animals of warm blood, as I have already had occafion to obferve (Sect. 26.), and particularly in the tongue.

I am, &c.

A. Volta.

*October* 25, 1792.

XII. *A Return*

